# Serum markers in interstitial pneumonia with and without *Pneumocystis jirovecii *colonization: a prospective study

**DOI:** 10.1186/1471-2334-9-47

**Published:** 2009-04-22

**Authors:** Yasuo Shimizu, Noriaki Sunaga, Kunio Dobashi, Makoto Fueki, Naoto Fueki, Sohei Makino, Masatomo Mori

**Affiliations:** 1 Department of Medicine and Molecular Science, Gunma University Graduate School of Medicine, 3-39-15 Showa-machi, Maebashi Gunma 371-8511, Japan; 2 Gunma University School of Health Sciences, 3-39-15 Showa-machi, Maebashi Gunma 371-8511, Japan; 3 Jobu Hospital for Respiratory Disease 586-1 Taguchi-machi, Maebashi, Gunma 371-0048, Japan; 4 WHO Collaborating Center of Prevention and Control of Chronic Respiratory Diseases, Dokkyo University, (DU-WCC) 880 Kitakobayashi Mibu Shimotsuga-gun, Tochigi 321-0293, Japan

## Abstract

**Background:**

In patients with chronic respiratory disease, *Pneumocystis jirovecii (P. jirovecii) *colonization is observed, and may influence disease progression and systemic inflammation. *Pneumocystis *pneumonia causes interstitial changes, so making a diagnosis of PCP in patients who have interstitial pneumonia (IP) with *P. jirovecii *colonization is sometimes difficult based on radiography.

**Methods:**

This study investigated the prevalence of *P. jirovecii *colonization in IP patients and assessed pulmonary injury due to *P. jirovecii *colonization by measurement of serum markers (KL-6, SP-A, SP-D, and (1→3) β-D-glucan (β-D-glucan)) and the peripheral lymphocyte counts, prospectively. A total of 75 patients with idiopathic pulmonary fibrosis (n = 29), collagen vascular-related interstitial pneumonia (n = 19), chronic bronchitis or pneumonia (n = 20), and *Pneumocystis *pneumonia (n = 7) were enrolled in this prospective study. *P. jirovecii *DNA was detected in sputum samples, while serum markers and the lymphocyte count were measured in the peripheral blood.

**Results:**

IP patients (idiopathic pulmonary fibrosis and collagen vascular-related IP) who received oral corticosteroids had a high prevalence of *P. jirovecii *colonization (23.3%). In IP patients, oral corticosteroid therapy was a significant risk factor for *P. jirovecii *colonization (*P *< 0.05). Serum markers did not show differences between IP patients with and without *P. jirovecii *colonization. The β-D-glucan level and lymphocyte count differed between patients with *Pneumocystis *pneumonia or *P. jirovecii *colonization.

**Conclusion:**

Serum levels of KL-6, SP-A, SP-D, and β-D-glucan were not useful for detecting *P. jirovecii *colonization in IP patients. However, the serum β-D-glucan level and lymphocyte count were useful for distinguishing *P. jirovecii *colonization from *pneumocystis *pneumonia in IP patients.

## Backgrounds

*Pneumocystis jirovecii *(*P. jirovecii*) is a fungus, and a high prevalence of *P. jirovecii *colonization has been reported among non-human immunodeficiency virus (HIV)-infected immunocompetent patients with primary respiratory disease [1-3]. Interstitial pneumonia (IP) is also associated with a high colonization rate of *P. jirovecii *[4]. The mortality rate of patients with acute exacerbation of IP is 78% after hospital admission [5]. After acute exacerbation of IP occurs in IP patient with *P. jirovecii *colonization, it is difficult to distinguish IP associated with *Pneumocystis *pneumonia (PCP) from IP without PCP based on the clinical and radiological features. When *P. jirovecii *is detected in sputum or bronchoalveolar lavage (BAL) fluid from IP patients with exacerbation, the clinician has to determine whether the patient has infection or colonization by *P. jirovecii*, and whether treatment should include sulfamethoxazole/trimethoprim (ST). Serum levels of KL-6 [6], surfactant protein A (SP-A), and surfactant protein D (SP-D) are used as markers of active IP [7]. KL-6 is an epithelial mucin and also membrane glycoprotein classified as cluster 9. KL-6 is more prominent in injured type II pneumocytes, and leaked into blood flow [6,8]. SP-A and SP-D are calcium-dependent lectins, and prominently produced by type II pneumocytes and Clara cells in injured lung [9]. PCP infection leads the accumulation of neutrophils and CD8 lymphocytes, and elicits the inflammatory mediator of macrophage inflammatory protein (MIP)-2, interleukin (IL)-8, and tumor necrosis factor (TNF)-α [10,11]. Mechansims of elevation of serum markers in PCP are thought that these mediators induce type II pneumocytes damages or hyperplasia, and this affects the elevations of KL-6, SP-A and SP-D on serum levels. Although elevation of serum levels of KL-6 and SP-A are reported in PCP, while elevation of the serum (1→3) β-D-glucan (β-D-glucan) level has been reported as a marker of PCP [12,13], whether these markers are affected by *P. jirovecii *colonization has not yet been investigated in IP patients. In the present study, we prospectively examined colonization by *P. jirovecii *in IP patients, including those with idiopathic pulmonary fibrosis (IPF) or collagen vascular disease-related interstitial pneumonia (CVD), and also examined *P. jirovecii *in patients with chronic bronchitis or pneumonia (CB/Pneumonia). Furthermore, we compared the levels of these serum markers between IP patients with or without *P. jirovecii *colonization to determine whether the markers were affected by colonization.

## Methods

### Subjects

A total of 75 patients were enrolled in this prospective study. The IPF group (n = 29, men/women = 18/11, age = 64.7 ± 10.2 years) included 22 patients with usual interstitial pneumonia (UIP) and 7 patients with non-specific interstitial pneumonia (NSIP). The CVD group (n = 19, men/women = 10/9, age = 63.6 ± 7.8 years) included 11 patients with rheumatoid arthritis, 3 with polymyositis, 2 with polyarteritis nodosa, 1 with dermatomyositis, 1 with mixed connective tissue disease, and 1 with Sjögren syndrome. The CB/Pneumonia group (n = 20) included 10 men and 10 women aged 66.8 ± 9.5 years. The PCP group (n = 7, men/women = 4/3, age = 61.6 ± 10.8 years) included 1 patient with ulcerative colitis, 3 with lung cancer, 1 with adrenal deficiency, 1 with human immunodeficiency virus, and 1 with polymyositis. The numbers of patients who received the oral corticosteroid therapy were 11 patients in UIP, 5 patients in NSIP, 14 patients in CVD and 1 patient in PCP. The diagnosis of IPF was made by using the American Thoracic Society/European Respiratory Society (ATS/ERS) International Multidisciplinary Consensus Classification of Idiopathic Interstitial Pneumonias [14]. PCP was diagnosed from the detection of *P. jirovecii *DNA in sputum or BAL by PCR, typical radiographic features, typical symptoms [15], and the course of chest symptoms and pulmonary changes on CT or chest radiographs. A diagnosis of *P. jirovecii *colonization was based on the detection of *P. jirovecii *DNA in induced sputum by PCR, no changes of chest X-ray films or chest CT scans for two months, no change of oxygen saturation for two months, no past or current PCP infection or HIV infection, no treatment with ST for PCP, and no clinical signs of PCP [16]. Patients receiving oral corticosteroids at doses above 5 mg/day were defined as the IP patients on corticosteroid therapy. This study was conducted according to the guidelines of the Declaration of Helsinki and approved by institutional review board. All of the patients are given written informed consent.

### Laboratory tests

Induced sputum samples were collected after inhalation of ultrasonically nebulized hypertonic saline. Sputum samples were subjected to PCR according to the method of Wakefield et al [17,18] using AmpliTaq DNA polymerase (Roche Diagnostics, Switzerland) and a PJ-2000 thermal cycler (Perkin Elmer, U.S.A). A peripheral blood sample was collected, and the lymphocyte count, CD4 T cell count, and serum KL-6 level were measured as described previously [19]. SP-A and SP-D were measured by using the SP-A test Kokusai F kit (Sysmex, Japan) and SP-D Yamasa EIA kit (Yamasa, Japan), respectively [7,20]. β-D-glucan was measured by the β-D-glucan test (Wako, Japan) based on a kinetic turbidmetric assay [21].

### Statistics

Categorical data were compared by using the chi-squared test or Fisher's exact test. The significance of differences between groups was calculated by Dunnet's test after the Kruskal-Wallis test. The relation of oral corticosteroid therapy (odds ratio) to *P. jirovecii *colonization was calculated by using the chi-squared test for comparison between IP patients receiving oral corticosteroids and those not on corticosteroid therapy. The significance of differences between IP patients on oral corticosteroid therapy with *P. jirovecii *colonization and IP patients on corticosteroid therapy without *P. jirovecii *colonization was calculated by the Man-Whitney test. Statistical significance was defined as *P *<0.05.

## Results

### The rate of P. jirovecii colonization on pulmonary diseases

**Figure 1:**
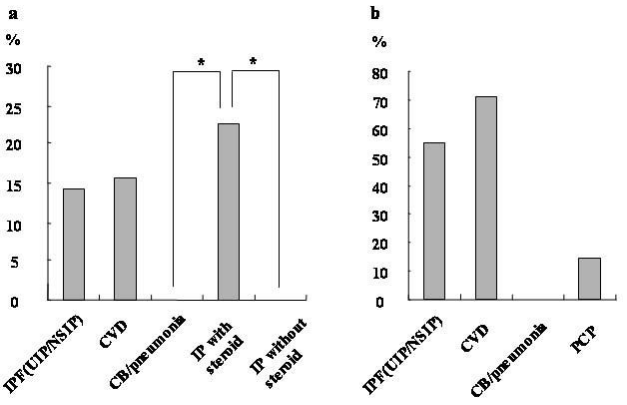
***P. Jirovecii *colonization rate in the including idiopathic pulmonary fibrosis (IPF), collagen vascular-related IP (CVD), and chronic bronchitis (CB)/pneumonia (pneumo) groups**. Patients with IP (IPF and CVD) were divided into groups receiving oral corticosteroid therapy and without corticosteroid therapy (a). The rates of oral corticosteroids use in IPF, CVD, CB/pneumonia and *Pneumocystis *pneumonia (PCP) group (b).

The *P. jirovecii *colonization rates were 13.8% in the IPF group and 15.8% in CVD group, while it was 0% in the CB/Pneumonia group. The *P. jirovecii *colonization rate of IP patients on oral corticosteroid therapy (n = 30), i.e., IPF and CVD patients on corticosteroids with *P. jirovecii *colonization, was significantly higher (23.3%) than the rate (0%) for IP patients (n = 18) who were not on corticosteroids (Fig. 1a). The odds ratio for *P. jirovecii *colonization in IP patients receiving oral corticosteroids was 16.3 (*P *= 0.0097). The use of oral corticosteroids was high in patients with IPF (55.2%) and CVD (73.7%), and the rate was not significantly different between the IPF group and the CVD group. None of the CB/pneumonia patients were on oral corticosteroid therapy, and only one patient was receiving oral corticosteroids in the PCP group (Fig. 1b).

### Serum marker levels and lymphocytes count in pulmonary diseases

**Figure 2:**
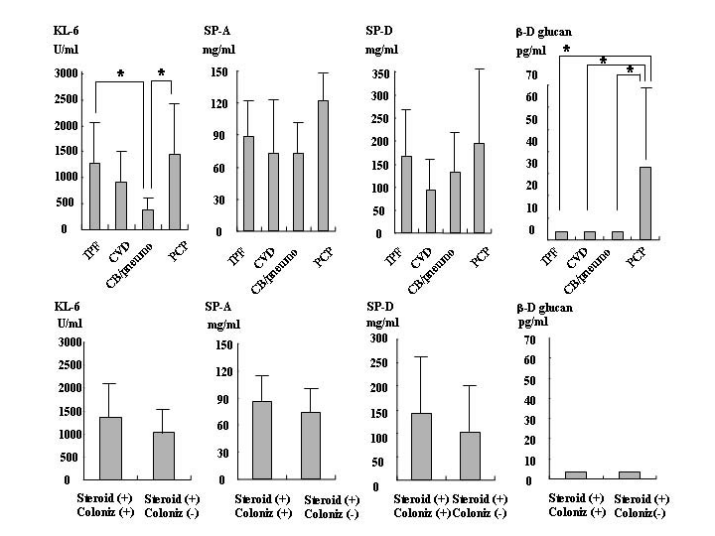
**Serum markers levels in pulmonary diseases**. Serum levels of KL-6, SP-A, SP-D, and β-D glucan in patients with respiratory diseases of idiopathic pulmonary fibrosis (IPF), collagen vascular-related IP (CVD), chronic bronchitis (CB)/pneumonia (pneumo) and pneumocystis pneumonia (PCP) groups. Levels of KL-6, SP-A, SP-D, and β-D glucan in IP patients on oral corticosteroids with *P. jirovecii *colonization (steroid (+)) colniz (+)) versus patients without *P. jirovecii *colonization (steroid (+)) colniz (-)).

**Figure 3:**
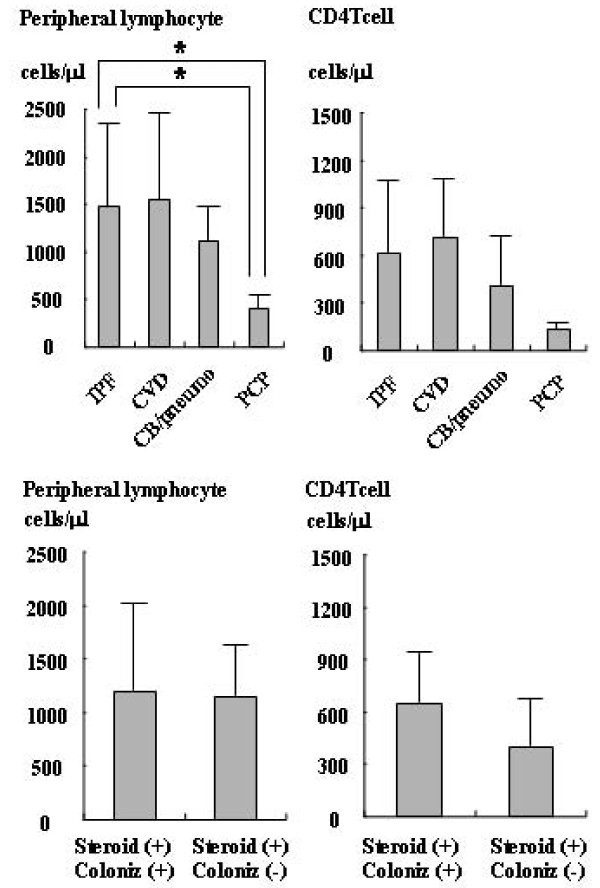
**Peripheral lymphocyte count and CD4 T cell count in respiratory diseases**. Peripheral lymphocyte count and CD4 T cell count in idiopathic pulmonary fibrosis (IPF), collagen vascular-related IP (CVD), chronic bronchitis (CB)/pneumonia (pneumo) and pneumocystis pneumonia (PCP) groups. Peripheral lymphocyte count and CD4 T cell count in IP patients on oral corticosteroid therapy with *P. jirovecii *colonization versus those without colonization.

Serum levels of KL-6 were higher in the IPF (1278.5 ± 740.4) and PCP (1488 ± 1086.1) groups than in the CB/Pneumonia group (375.8 ± 300.8). The KL-6 level of the CVD group (898.2 ± 605.3) was not different from that of the PCP group (Fig. 2a). SP-A levels did not show significant differences among the IPF group (88.7 ± 36.2), CVD group (73.2 ± 52.1), CB/Pneumonia group (73.3 ± 41.4), and PCP group (121.9 ± 24.7) (Fig. 2b). SP-D levels also did not show significant differences among the IPF group (166.9 ± 111.1), CVD group (92.7 ± 67.7), CB/Pneumonia group (131 ± 71.0), and PCP group (194.1 ± 157.5) (Fig. 2c). The β-D-glucan level of the PCP (32.8 ± 35.1) was significantly higher than that of the other groups (the IPF, CVD, and CB/pneumonia groups all had levels under the detection limit of <3.4) (Fig. 2d). Levels of serum markers did not show any significant differences between IP patients with and without *P. jirovecii *colonization on oral corticosteroid therapy with respect to KL-6 (1370 ± 810.4 vs. 1030.6 ± 594.6), SP-A (86.6 ± 32.8 vs. 73.5 ± 44.5), SP-D (142.8 ± 145.1 vs. 102.3 ± 121.9), and β-D-glucan (both groups were under the detection limit) (Figs. 2e, f, g and 2h). The lymphocyte count of the PCP group (397.1 ± 151.6) was significantly lower than those of the other groups (IPF: 1480.2 ± 917.1; CVD: 1543.8 ± 919.6; and CB/pneumonia: 1118.4 ± 445.3). CD4 T cell counts showed no statistical differences among the four groups (IPF: 614.8 ± 469; CVD: 711.8 ± 431.1; CB/pneumonia: 403.3 ± 369.9; and PCP: 129 ± 96.7) (Figs. 3a, b). The lymphocyte count and CD4 T cell count showed no statistical differences between IP patients with and without *P. jirovecii *colonization on oral corticosteroids (Figs. 3c, d). The peripheral lymphocyte count of the former group was 1206.7 ± 859.7 and that of the latter group was 1144.6 ± 560.0, while the CD4 T cell count was 651.6 ± 565.6 and 401.5 ± 235.4, respectively.

## Discussion

*P. jirovecii *colonization occurs in patients with chronic respiratory disease, even in the absence of HIV infection [22]. The *P. jirovecii *colonization rate is 15.5% in pregnant women [23] and 10% to 40% in chronic obstructive disease (COPD) patients [1,24,25]. In the present study, IP patients who received oral corticosteroid therapy had a high *P. jirovecii *colonization rate of 23.3%. Oral steroid therapy was a statistical risk factor for *P. jirovecii *colonization. Furthermore, the serum β-D-glucan level and the lymphocyte count were useful to distinguish IP with *P. jirovecii *colonization patients from PCP patients, while serum SP-A, SP-D, and KL-6 levels were not useful. These serum markers were not affected by *P. jirovecii *colonization.

β-D-glucan is a major component of the *P. jirovecii *cyst wall [26]. Infection is established by adhesion of the trophic form of *Pneumocystis *to the alveolar epithelium, where it proliferates [27]. One of the reasons for the different levels of β-D-glucan in PCP and *P. jirovecii *colonization was considered to be *Pneumocystis *proliferation. Cyst wall β-D-glucan is partly responsible for the inflammatory response in the lung [28,29]. SP-A and SP-D levels were reported to be affected by PCP infection via inflammatory mediators [10,11] or reported to influence on PCP infection, and to play a role in host defenses against *P. jirovecii *[30-32]. Based on these reports, we examined serum KL-6, SP-A, and SP-D levels in IP patients with or without *P. jirovecii *colonization on oral corticosteroids. Our results indicated that these markers did not show any difference between IP patients with and without *P. jirovecii *colonization, suggesting that KL-6, SP-A, and SP-D levels were not affected by *P. jirovecii *colonization in IP patients. Although there has been a report that *P. jirovecii *colonization plays a role in systemic inflammation in COPD patients [33], we did not examine the effect of *P. jirovecii *on other cytokines or on disease progression. To determine the effect of *P. jirovecii *colonization on serum markers, it would be necessary to compare the changes of each marker when new *P. jirovecii *colonization occurs in a patient. Since present study did not show the affect of *P. jirovecii *colonization on serum markers of KL-6, SP-A and SP-D, we could not determine the value of prophylaxis with ST for IP patients with *P. jirovecii *colonization against pulmonary injury. There are unsolved problems regarding droplet transmission of *P. jirovecii *and the existence of drug-resistant organisms associated with prophylaxis of ST. Further analysis will be needed to determine the efficacy of ST prophylaxis for IP patients with *P. jirovecii *colonization on oral corticosteroid therapy.

Recently, quantification of *P. jirovecii *DNA by real-time PCR targeting the heat shock protein 70 gene was reported to be useful for discrimination between colonization and infection with this organism [34]. When *P. jirovecii *is detected in sputum or bronchoalveolar lavage (BAL) fluid from a patient with exacerbation of IP, the clinician has to determine whether a patient has infection or colonization. To make a diagnosis of PCP, real-time PCR of the HSP 70 gene might be used in the clinical setting.

## Conclusion

In conclusion, serum markers (KL-6, SP-A, and SP-D) were not higher in IP patients with *P. jirovecii *colonization than in those without colonization. IP patients receiving oral corticosteroid therapy had a high prevalence of *P. jirovecii *colonization, and oral corticosteroids are a risk factor for colonization. When patients develop exacerbation of IP, to diagnosis PCP or *P. jirovecii *colonization, it seems to be useful to know in advance whether the patient is a carrier of *P. jirovecii*, and to also examine the serum β-D-glucan level and the peripheral lymphocyte count.

## Competing interests

The authors declare that they have no competing interests.

## Authors' contributions

Yasuo Shimizu MD., PhD: Design, Gathering Data, Statistical Analysis and Preparation of manuscript, Noriaki Sunaga MD., PhD: Gathering Data, Statistical Analysis, Kunio Dobashi MD., Prof: Reviewing Manuscript, Makoto Fueki MD: Gathering Data, Naoto Fueki MD., PhD: Gathering Data, Sohei Makino MD., Prof: Reviewing Manuscript, Masatomo Mori MD., Prof: Reviewing Manuscript. All authors read and approved the final manuscript.

## Pre-publication history

The pre-publication history for this paper can be accessed here:

http://www.biomedcentral.com/1471-2334/9/47/prepub
